# Severe Maternal Sepsis in the UK, 2011–2012: A National Case-Control Study

**DOI:** 10.1371/journal.pmed.1001672

**Published:** 2014-07-08

**Authors:** Colleen D. Acosta, Jennifer J. Kurinczuk, D. Nuala Lucas, Derek J. Tuffnell, Susan Sellers, Marian Knight, on behalf of the United Kingdom Obstetric Surveillance System

**Affiliations:** 1 National Perinatal Epidemiology Unit, University of Oxford, Oxford, United Kingdom; 2 Department of Anaesthesia, Northwick Park Hospital, Harrow, United Kingdom; 3 Bradford Royal Infirmary, Bradford Hospitals NHS Trust, Bradford, United Kingdom; 4 St. Michael's Hospital, University Hospitals Bristol NHS Trust, Bristol, United Kingdom; University of Queensland, Australia

## Abstract

**Background:**

In light of increasing rates and severity of sepsis worldwide, this study aimed to estimate the incidence of, and describe the causative organisms, sources of infection, and risk factors for, severe maternal sepsis in the UK.

**Methods and Findings:**

A prospective case-control study included 365 confirmed cases of severe maternal sepsis and 757 controls from all UK obstetrician-led maternity units from June 1, 2011, to May 31, 2012. Incidence of severe sepsis was 4.7 (95% CI 4.2–5.2) per 10,000 maternities; 71 (19.5%) women developed septic shock; and five (1.4%) women died. Genital tract infection (31.0%) and the organism *Escherichia coli* (21.1%) were most common. Women had significantly increased adjusted odds ratios (aORs) of severe sepsis if they were black or other ethnic minority (aOR = 1.82; 95% CI 1.82–2.51), were primiparous (aOR = 1.60; 95% CI 1.17–2.20), had a pre-existing medical problem (aOR = 1.40; 95% CI 1.01–1.94), had febrile illness or were taking antibiotics in the 2 wk prior to presentation (aOR = 12.07; 95% CI 8.11–17.97), or had an operative vaginal delivery (aOR = 2.49; 95% CI 1.32–4.70), pre-labour cesarean (aOR = 3.83; 95% CI 2.24–6.56), or cesarean after labour onset (aOR = 8.06; 95% CI 4.65–13.97). Median time between delivery and sepsis was 3 d (interquartile range = 1–7 d). Multiple pregnancy (aOR = 5.75; 95% CI 1.54–21.45) and infection with group A streptococcus (aOR = 4.84; 2.17–10.78) were associated with progression to septic shock; for 16 (50%) women with a group A streptococcal infection there was <2 h—and for 24 (75%) women, <9 h—between the first sign of systemic inflammatory response syndrome and a diagnosis of severe sepsis. A limitation of this study was the proportion of women with sepsis without an identified organism or infection source (16.4%).

**Conclusions:**

For each maternal sepsis death, approximately 50 women have life-threatening morbidity from sepsis. Follow-up to ensure infection is eradicated is important. The rapid progression to severe sepsis highlights the importance of following the international Surviving Sepsis Campaign guideline of early administration of high-dose intravenous antibiotics within 1 h of admission to hospital for anyone with suspected sepsis. Signs of severe sepsis in peripartum women, particularly with confirmed or suspected group A streptococcal infection, should be regarded as an obstetric emergency.

*Please see later in the article for the Editors' Summary*

## Abstract 2

Marion Knight and colleagues conducted a national prospective case-control study in the UK from June 2011 through May 2012 to estimate the incidence, describe the causative organisms and sources of infection, and identify the risk factors for severe maternal sepsis.

*Please see later in the article for the Editors' Summary*

## Editors' Summary

**Background:**

Every year, nearly 300,000 women worldwide die during pregnancy or labour, or shortly after. According to a recent World Health Organization estimate, sepsis (blood poisoning) is responsible for 10.7% of these maternal deaths. Sepsis is caused by an inappropriate immune response to an infection. Normally, when bacteria or other microbes enter the human body, the immune system efficiently destroys the invaders. In sepsis, the immune system goes into overdrive, and the chemicals it releases into the blood to combat infection trigger widespread inflammation. This inflammation leads to the formation of small blood clots and leaky blood vessels that block the flow of blood to the vital organs. In the most severe cases (septic shock), blood pressure falls to dangerously low levels, multiple organs fail, and the patient can die. Symptoms of sepsis include fever, rapid breathing, and a fast heart rate. Sepsis, which often progresses rapidly, can be treated in its early stages with antibiotics alone. People with severe sepsis need to be admitted to an intensive care unit, where their vital organs can be supported while the infection is treated.

**Why Was This Study Done?:**

Deaths from maternal sepsis mainly occur in low- and middle-income countries, but the rate of such deaths is increasing in countries with advanced healthcare systems. In the UK, for example, the incidence (the number of cases) of fatal maternal sepsis has increased markedly over the past two decades, and although the absolute risk of maternal death from sepsis is low, increasing numbers of women are experiencing severe maternal sepsis. To avoid preventable maternal illness and death in the UK, it is essential that clinical management and infection control strategies for maternal sepsis are improved. Here, to learn more about the incidence of maternal sepsis, the causative organisms and sources of infection, and the risk factors for maternal sepsis in the UK, the researchers undertake a national case-control study of severe maternal sepsis. A case-control study compares the characteristics of individuals with and without a given disease.

**What Did the Researchers Do and Find?:**

For this study, clinicians in all the UK obstetrician-led maternity units (obstetricians care for women throughout pregnancy, labour, and the post-labour period) sent information about every woman who developed severe sepsis between June 2011 and May 2012 (365 cases) and about two unaffected (control) women per case to the United Kingdom Obstetric Surveillance System (UKOSS). Using this information and data on the number of maternities in the UK during this 12-month period, the researchers calculated that the incidence of severe sepsis was 4.7 per 10,000 maternities. Seventy-one women with severe sepsis (19.5% of cases) developed septic shock, and five women (1.4% of cases) died. The most common source of sepsis (implicated in about a third of cases) was a genital tract infection. Statistical analyses identified several risk factors for severe maternal sepsis, including having a fever or taking antibiotics in the two weeks preceding sepsis and all types of operative delivery (including cesarean delivery). Importantly, although *Escherichia coli* was the most common causative organism in severe maternal sepsis (present in a fifth of cases), infection with group A streptococcus was strongly associated with progression to septic shock. Moreover, in half the women with a group A streptococcal infection, severe sepsis was diagnosed within two hours of the first signs of a systemic inflammatory response.

**What Do These Findings Mean?:**

These findings show that for every death from maternal sepsis in the UK, about 50 women develop life-threatening severe sepsis, that the onset of severe sepsis is very rapid, and that women who have recently had an infection are at particularly high risk of developing maternal sepsis. Although some pregnant women who developed severe sepsis during the study period may not have been included in the study, these findings have important clinical implications for the management of maternal sepsis in the UK and elsewhere. The findings suggest that pregnant or recently pregnant women with an infection need closer attention than women who are not pregnant, and adequate follow-up to ensure eradication of the infection. The findings also highlight the importance of giving high-dose intravenous antibiotics to anyone with suspected sepsis within an hour of admission to hospital as recommended by the international Surviving Sepsis Campaign, an initiative that was developed to improve the management, diagnosis, and treatment of sepsis. Finally, these findings suggest that signs of severe sepsis, particularly in women with a confirmed or suspected group A streptococcal infection, should be regarded as an obstetric emergency.

**Additional Information:**

Please access these websites via the online version of this summary at http://dx.doi.org/10.1371/journal.pmed.1001672.

- The UK National Health Service Choices website has information about sepsis
- The international Surviving Sepsis Campaign guidelines for the treatment of sepsis are available through the campaign's website
- The Sepsis Alliance, a US not-for-profit organization, also provides information about sepsis for patients and their families (in English and Spanish), including information about maternal sepsis and several personal stories about maternal sepsis (see the stories of Alanna Basinger, Alisa Proctor, Sandy C, and Natalie Banathy)
- The not-for profit UK Sepsis Trust is another useful source of information about sepsis that includes patient stories
- MedlinePlus provides links to additional resources about sepsis (in English and Spanish)
- UKOSS provides more information about its national case-control study on severe maternal sepsis in the UK

## Introduction

Maternal death from sepsis is increasing in countries with advanced healthcare systems [1]–[4], and sepsis is estimated to cause 9.7%, 11.6%, and 7.7% of maternal deaths in Africa, Asia, and Latin America and the Caribbean, respectively [5]. Sepsis is now the leading cause of direct maternal death in the United Kingdom [2]. In 2006–2008, the UK maternal mortality rate from sepsis was 1.13/100,000 maternities, a rate not seen since the early 1970s [2],[6]. Underlying this trend is an increasing number of maternal deaths from group A streptococcal infection, most recently accounting for 50% of direct maternal sepsis deaths. This trend has also been observed in the Netherlands [7]. Although the absolute risk of maternal death from sepsis is low, an increase in maternal mortality implies a greater number of women with severe, life-threatening illness. Recent work has suggested an approximate doubling of the incidence of maternal sepsis in the US since 2003 [4].

Key information gaps in the understanding of this pressing problem are the number of women affected, causative organisms, sources of infection, and risk factors for severe sepsis and poor outcomes such as septic shock. Sepsis progresses along a spectrum of severity, so clarity about these factors has urgent implications for clinical management and infection control strategies to avoid preventable maternal deaths.

The objectives of this national prospective case-control study were to estimate the incidence, describe the causative organisms and sources of infection, and identify the risk factors for severe maternal sepsis in the UK. This information will inform strategies to improve outcomes for mothers and their babies through further development of guidelines for prevention and management of sepsis in pregnancy in the UK.

## Methods

### Research Ethics Committee Approval

This study was approved by the London Research Ethics Committee (ref 10/H0717/20).

### Study Design

We undertook a national prospective case-control study of all peripartum women diagnosed with severe sepsis (including septic shock), irrespective of the source of infection, together with control women, in all obstetrician-led maternity units in the UK from June 1, 2011, to May 31, 2012. All UK hospitals with obstetrician-led maternity units participated in the study (168 in England, nine in Northern Ireland, 16 in Scotland, 14 in Wales, three in the Crown Dependencies). The study included a descriptive analysis of the incidence, causative organisms, sources of infection, and outcomes of severe sepsis, and a case-control analysis of factors associated with severe sepsis and septic shock. In order to assess risk factors for developing severe sepsis, all cases were compared with non-septic controls. To assess the risk of progression to septic shock, cases with a diagnosis of septic shock were compared to all other cases with severe sepsis that did not develop into septic shock.

### Data Source and Definitions

This study was conducted using the United Kingdom Obstetric Surveillance System (UKOSS). The UKOSS methods have been described elsewhere [8]. In brief, the UKOSS network of collaborating clinicians includes up to four nominated reporting clinicians (obstetricians, midwives, anaesthetists, and risk managers) in each obstetrician-led maternity unit in the UK. Nominated clinicians coordinate case reports from all clinicians in their units, and for this study were asked to report, via a monthly report card, how many women met the case definition for severe sepsis. Clinicians were asked to return all cards, including those with no cases to report, in order for participation to be monitored. Clinicians who reported a case were then sent a data collection form with a unique UKOSS identification number, requesting further detailed information on obstetric and medical history, diagnosis, management, and outcomes. Reporting clinicians were also asked to complete a data collection form for two women meeting the control definition. All data collected were new, and not based on routinely collected hospital admissions data. If completed data collection forms were not returned, up to four further reminders were given (after 6 wk, a second form was sent out, and a third form 4 wk thereafter; if there was still no response after a further 4 wk, the clinician was contacted by telephone). Overall, UKOSS has a 93% card return rate [8]. Where data were missing or invalid, clinicians were contacted for the correct information. All data were double entered into a customised database, and cases were verified to ensure that they met the case definition and to exclude duplicate reports.

Since there is currently no standardised definition for severe sepsis in pregnant and peripartum women, the study definition was developed based on previous literature and by consensus of the UKOSS steering committee [8]. In the non-obstetric population, consensus definitions of sepsis severity (systemic inflammatory response syndrome [SIRS], sepsis, severe sepsis, and septic shock) were developed in 1992 (Box 1) [9]. These definitions and subsequent improvements, however, are often not applicable to pregnant and peripartum women since clinical signs and symptoms of severe infection differ in this population. Specifically, SIRS can be a sign of ruptured membranes and changing biochemistry associated with labour and delivery, as well as a clinical marker of severe infection. Therefore, the clinical parameters of SIRS in the presence of an infection are often altered in the obstetric population. We adopted the “obstetric SIRS” criteria from a 2001 study of severe obstetric morbidity [1] and took into account clinical management (level 2 or level 3 critical care [10]) and whether the woman died. The full case definition for this study is listed in Box 1. Controls were women who did not have severe sepsis and delivered immediately before each case in the same hospital. For women transferred to higher-level hospitals, controls were drawn from the delivery hospital. The source population was thus all women giving birth in the UK.

**Box 1. General Sepsis Definitions and Study Definition of Severe Sepsis**

General Sepsis Definitions*

**SIRS**—Two of the following: temperature >38°C or <36°C, heart rate >90 beats/min, respiratory rate >20 breaths/min, or PaCO_2_ <32 mmHg (4.3 kPa), white cell count >12,000 cells/µl or <4,000 cells/µl, or 10% immature/band forms.

**Sepsis—**SIRS with infection.

**Severe sepsis—**Sepsis associated with organ dysfunction, hypoperfusion, or hypotension. Hypoperfusion and perfusion abnormalities may include, but are not limited to, lactic acidosis, oliguria, or an acute alteration in mental status.

**Septic shock—**Sepsis associated with hypotension, despite adequate fluid resuscitation, along with the presence of perfusion abnormalities as listed for severe sepsis. Patients who are on inotropic or vasopressor agents may not be hypotensive at the time that perfusion abnormalities are measured.

**Study Definition of Severe Sepsis**

Applied to women at any point in pregnancy and up to 6 wk postpartum:

1. Death related to infection or suspected infection
2. Any woman requiring level 2 or level 3 critical care (or obstetric high-dependency unit–type care) with severe sepsis or suspected severe sepsis
3. A clinical diagnosis of severe sepsis:
  - Temperature >38°C or <36°C, measured on two occasions at least 4 h apart
  - Heart rate >100 beats/min, measured on two occasions at least 4 h apart
  - Respiratory rate >20/min, measured on two occasions at least 4 h apart
  - White cell count >17×10^9^/l or <4×10^9^/l or with >10% immature band forms, measured on two occasions

*Source: 1992 American College of Chest Physicians/Society of Critical Care Medicine definitions [9].

Level 2 care is defined as patients requiring more detailed observation or intervention, single failing organ system, or postoperative care, and higher levels of care. Level 3 care is defined a patients requiring advanced respiratory support alone or basic respiratory support together with support of at least two organ systems. This level includes all complex patients requiring support for multi-organ failure. [10]

### Statistical Analyses

Stata statistical software 11 (StataCorp) was used for all analyses. The incidences of severe maternal sepsis and septic shock with 95% confidence intervals were calculated using the number of maternities reported in the most recent national birth data (2011) [11]–[13] as the denominator, since data are not available on the actual population at risk (number of women who have had a pregnancy, including women who have had miscarriages or pregnancy terminations). In these data, a maternity is defined as any woman giving birth to a live or stillborn infant of greater than 24 completed weeks of gestation. Women with signs and symptoms of sepsis prior to delivery were classified as an antepartum cases. Sources of infection, causative organisms, and sepsis severity characteristics were tabulated for all cases, and stratified according to partum status, as pathogenesis is known to differ between pregnant and postpartum women [14]. Groups were compared using a chi-square test for categorical variables; corresponding *p*-values are reported in the text.

For risk factor analyses, sociodemographic, medical history, and delivery characteristics with a priori evidence of an association with sepsis were compared between cases and controls, and between cases with and without septic shock. Sources of infection and causative organisms were also assessed as risk factors in the latter comparison. Comparisons were made using Pearson's chi-square and Fisher's exact tests where appropriate. All *p*-values were two-sided, and a *p*-value of <0.05 was considered statistically significant. The proportion of missing data in this study was very low; the only variables with substantial missing data (>1%) were source and organism of infection, and socio-economic group. It is common to have sepsis patients without a clear source of infection and/or cultures that are negative [15], and a previous UKOSS study found that women with unknown socio-economic information had significantly higher odds of severe maternal morbidity [16]. It is not likely therefore that missing data for these variables were missing at random, and thus a missing data technique such as multiple imputation would not have been appropriate. In order to account for the missing data for sources of infection, causative organisms, and socio-economic group, the subcategories of “unknown” and “no laboratory-confirmed infection” were included for these variables in all analyses.

The odds of severe sepsis and septic shock associated with each risk factor were estimated using univariable unconditional logistic regression and were then adjusted using multivariable unconditional logistic regression. (Since convenience matching was used, and thus the cases and controls were not matched according to criteria relevant to the analysis, conditional logistic regression was not needed [17].) For both the severe sepsis and septic shock outcome groups, factors were adjusted in two stages. First, all a priori sociodemographic and medical history factors, with the exception of previous cesarean delivery and previous pregnancy problem (as these were dependent on parity) and partum status (since the control population was only women who had delivered), were included in a primary model. Second, delivery factors were then adjusted for a priori risk factors using a more parsimonious approach in order to avoid overadjustment or substantial colinearity given the large number of variables; results were adjusted only for a priori factors from the primary model that were known risk factors, were significant in the primary model at *p*<0.05, or were plausible confounders as identified in previous literature. Delivery characteristics were evaluated for postpartum cases only, as this set of risk factors pertained specifically to delivery.

In the multivariable models, major pre-existing medical problems and complications of delivery were first included into the models as separate categories in order to check the significance of any conditions expected to have an association with severe sepsis. No significant differences in individual conditions between cases and controls were identified. As the numbers of individual pre-existing medical problems and complications of delivery were very small—with subsequent insufficient power to confidently detect statistical differences between cases and controls for these small groups—individual conditions were combined into aggregate variables. Diabetes, history of pyelonephritis/urinary tract infection, and history of sexually transmitted infection were retained as separate categories because these factors have been cited as independent risk factors for sepsis [4],[18].

Results of both stages of adjustment are reported as unadjusted odds ratios (uORs) and adjusted odds ratios (aORs) and their 95% CIs for severe sepsis. For ease of presentation of risk factors for progression to septic shock, results are reported only for factors included in the final adjusted models. Likelihood ratio tests with a significance level of *p*<0.01 were used to check for interactions between variables; no significant interactions were identified in the final adjusted models.

### Sample Size and Power

Within a 1-y study period, we anticipated approximately 316 cases of severe sepsis based on an estimated incidence of four per 10,000 maternities [1]. For the severe sepsis risk factor analysis, with two controls per case, and for a risk factor prevalence of at least 5% in control women, the study was estimated to have had 80% power at *p*<0.05 (two-sided) to detect a statistically significant odds ratio (OR) of 2.3 or greater. The actual number of cases and controls identified during the study period of 12 mo generated an estimated power of 80% at the 5% level of significance to detect an OR of 2.1 or greater, for the same risk factor prevalence level. For the septic shock risk factor analysis, for a risk factor prevalence of at least 15% in women without septic shock, the analysis had 80% power at the 5% level of significance to detect an OR of 2.6 or greater.

## Results

### Incidence

**Figure 1:**
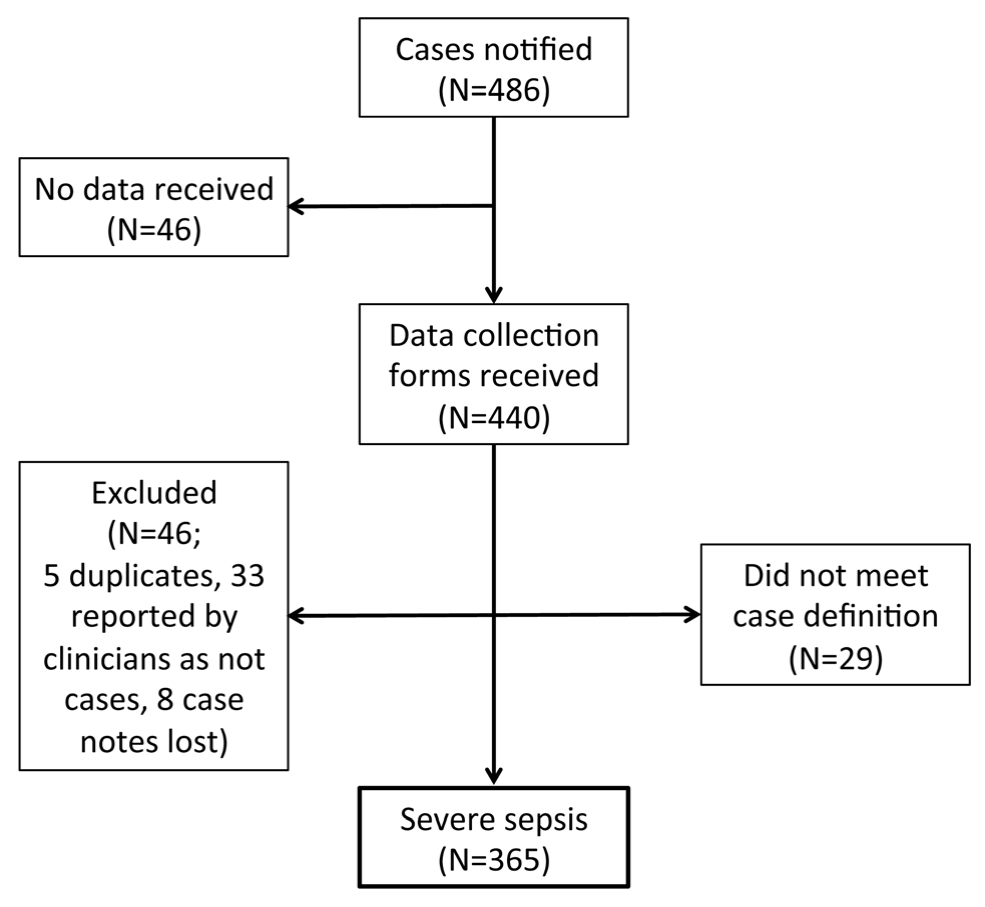
Case reporting and completeness of data collection.

During the study period, all 214 UK hospitals with obstetrician-led maternity units participated in UKOSS, representing 100% participation. There were a total of 486 cases of severe sepsis reported, of which data collection was complete for 90% (Figure 1), and data were obtained for 757 controls. Of the reported cases, 29 did not meet the case definition and were excluded from the study; of these 29 cases, 11 had only one control form returned, and 20 control forms had incomplete data and were thus excluded, leaving 27 additional controls that were included in the study. There was a total of 365 confirmed cases of severe sepsis out of 780,537 maternities in the UK [11]–[13], representing an incidence of 4.7 per 10,000 maternities (95% CI 4.2–5.2). Seventy-one women (20%) developed septic shock, which represents an incidence of 0.91 per 10,000 maternities (95% CI 0.71–1.15).

### Sources, Causative Organisms, and Severity

**Figure 2:**
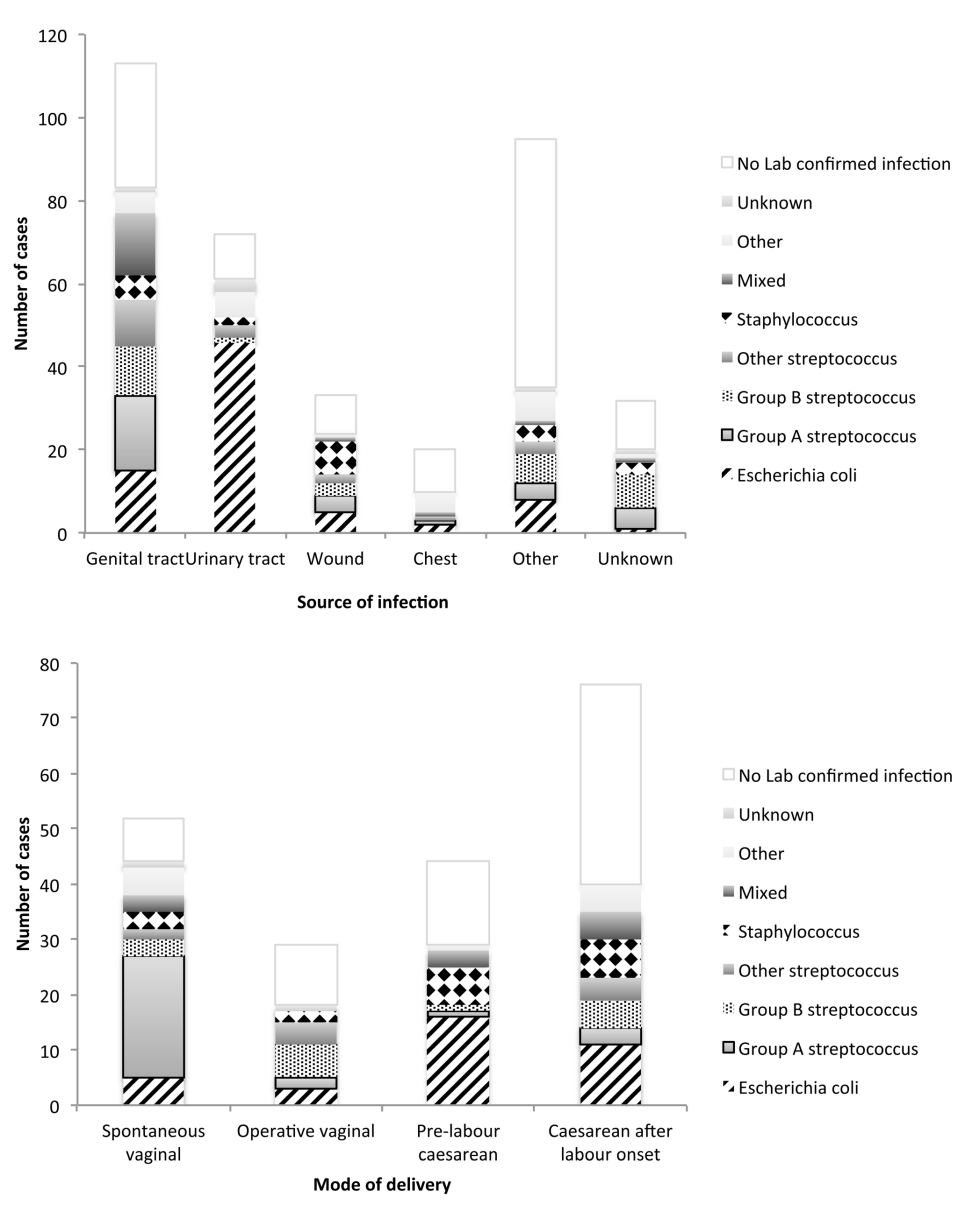
Distribution of causative organisms according to source of infection and mode of delivery. Stacked bars represent the number of cases with specific causative organisms according to infection source and mode of delivery categories. Data are mutually exclusive.

Laboratory-confirmed infection was reported for 233 (63.8%) severe sepsis cases, and a source of infection was identified for 270 cases (74.0%); 60 cases (16.4%) had neither a source of infection or causative organism identified. The distribution of sources of infection, causative organisms, and severity characteristics are shown in Table 1 and Figures 1 and 2. Overall, the largest proportion of cases was due to genital tract infection (31.0%), and the most common organism causing infection was *Escherichia coli* (21.1%). However, the distributions of both the infection source and the causative organism differed significantly between women with antepartum versus postpartum sepsis (*p*<0.0001 for both), as did the risk of septic shock. Readmission (for reasons other than delivery) also differed significantly between the two groups; 108 (48%) women with postpartum sepsis were readmitted, compared to six (5%) women with antepartum sepsis (*p*<0.0001). Of all cases, 286 (78%) received level 2 or intensive care, and five women died (Table 1). Of the women who died, two had infection with *E. coli*, and three women had an unknown causative organism. Twenty-nine (8%) women with severe sepsis had either a miscarriage or a termination of pregnancy. For women diagnosed with severe sepsis antenatally, five of 137 infants were stillborn (3.6%), and seven died in the neonatal period (5.1%). Fifty-eight infants (42.3%) were admitted to neonatal intensive care.

**Table 1:** Characterisitcs of infection in women with severe antepartum and postpartum sepsis.

Characteristic	Antepartum *n* (Percent)	Postpartum* *n* (Percent)	Chi-Square *p*-Value	Total *n* (Percent)
**All**	134 (36.7)	231 (63.3)		365 (100)
**Source of infection**			<0.0001	
Genital tract	27 (20.2)	86 (37.2)		113 (31.0)
Urinary tract	45 (33.6)	27 (11.7)		72 (19.7)
Wound	0 (0.0)	33 (14.3)		33 (9.0)
Respiratory	12 (9.0)	8 (3.5)		20 (5.5)
Other	10 (7.5)	22 (9.5)		32 (8.8)
Unknown	40 (29.9)	55 (23.8)		95 (26.0)
**Organism**			<0.0001	
*E. coli*	33 (24.6)	44 (19.1)		77 (21.1)
Group A streptococcus	2 (1.5)	30 (13.0)		32 (8.8)
Group B streptococcus	13 (9.7)	17 (7.4)		30 (8.2)
Other streptococcus	6 (4.5)	15 (6.5)		21 (5.7)
Staphylococcus	2 (1.5)	21 (9.1)		23 (6.3)
Mixed organisms	5 (3.7)	14 (6.1)		19 (5.2)
Other	12 (9.0)	13 (5.6)		25 (6.9)
Unknown	5 (3.7)	1 (0.4)		6 (1.6)
No laboratory-confirmed infection	56 (41.8)	76 (32.9)		132 (36.2)
**Severity**				
Level 2 or ICU admission	103 (76.9)	183 (79.2)	0.598	286 (78.4)
Level 2 admission	64 (47.8)	107 (46.3)	0.79	171 (46.9)
ICU admission**	39 (29.1)	75 (32.5)	0.504	114 (31.2)
Septic shock	16 (11.9)	55 (23.8)	0.006	71 (19.5)
Death	2 (1.5)	3 (1.3)	0.915	5 (1.4)

*Includes women with sepsis after first/second trimester losses (*n* = 29).

**Irrespective of level 2 admission.

ICU, intensive care unit.

### Time Course

The median gestational age at antenatal sepsis diagnosis was 35 wk (interquartile range [IQR] 27–40 wk). The median diagnosis-to-delivery interval for women with antenatal sepsis was 0 d (IQR 0–36 d). The median time between delivery and sepsis for postpartum cases was 3 d (IQR 1–7 d). There were 296 cases with recorded dates and times for the first sign of SIRS and the severe sepsis diagnosis; for 245 (83%) severe sepsis cases and for 49 (85%) septic shock cases, there was <24 h between the first sign of SIRS and the diagnosis of severe sepsis; and for 264 (89%) severe sepsis cases and for 55 (95%) septic shock cases there was <48 h between the first sign of SIRS and the diagnosis of severe sepsis. For 95 (86%) women who were readmitted there was <24 h between the first sign of SIRS and diagnosis of severe sepsis. Additionally, for 16 (50%) women with a group A streptococcal infection there was <2 h—and for 24 (75%) women <9 h—between the first sign of SIRS and the diagnosis of severe sepsis.

### Risk Factors for Severe Sepsis

A priori sociodemographic and medical history characteristics of women with severe sepsis compared to control women are listed in Table 2. After adjustment and compared to controls, women who were of black or other minority ethnic origin, were primiparous, had a pre-existing medical problem, or had a febrile illness or were taking antibiotics in the 2 wk prior to presentation were at significantly increased odds of severe sepsis. There was no statistically significant association between premature rupture of membranes and severe sepsis in either antenatal cases (*n* = 20; aOR = 1.72; 95% CI 0.98–3.02) or postnatal cases (Table 3). In addition to significant a priori factors, the following factors significantly increased the odds of severe sepsis in women with postpartum sepsis: having an operative vaginal delivery (aOR = 2.49; 95% CI 1.32–4.70), having a pre-labour cesarean section (aOR = 3.83; 95% CI 2.24–6.56) or a cesarean section after the onset of labour (aOR = 8.06; 95% CI 4.65–13.97), or having a complication of delivery (aOR = 1.69; 95% CI 1.09–2.63) (Table 3). Of note, of all women who had a cesarean section, 96.6% of cases and 94.8% of controls received prophylactic antibiotics at delivery.

**Table 2:** Unadjusted and adjusted odds ratios for severe sepsis associated with sociodemographic and medical factors; all severe sepsis cases compared with controls.

Category	Risk Factor	Cases *n* (Percent)*, *n* = 365	Controls *n* (Percent)*, *n* = 757	Chi-Square *p*-Value	uOR	95% CI	aOR**	95% CI
**Sociodemographic factors**	**Age (years)**			<0.001				
	<25	117 (32.0)	158 (20.9)		1.73	1.29–2.32	1.38	0.96–2.00
	25–34	186 (51.0)	438 (57.9)		1		1	
	≥35	62 (17.0)	160 (21.1)		0.91	0.65–1.28	1.00	0.67–1.51
	**Ethnic group**			0.003				
	White	221 (60.7)	525 (69.5)		1		1	
	Black and other minority	143 (39.3)	230 (30.5)		1.48	1.14–1.92	1.82	1.32–2.51
	**Socio-economic group** ***			0.001				
	Managerial and professional occupations	68 (19.7)	189 (25.6)		1		1	
	Intermediate	63 (18.2)	147 (20.0)		1.19	0.79–1.79	1.17	0.73–1.88
	Manual	96 (27.8)	224 (30.4)		1.19	0.83–1.72	1.26	0.81–1.94
	Unemployed	29 (8.0)	46 (6.1)		1.75	1.02–3.01	1.56	0.82–2.97
	Unknown	109 (29.9)	151 (19.9)		2	1.38–2.91	1.63	1.02–2.61
	**Marital status**			0.006				
	Single	85 (23.3)	124 (16.4)		1.54	1.13–2.10	1.13	0.75–1.69
	Married or cohabitating	280 (76.7)	630 (83.6)		1		1	
**Obstetric and medical factors**	**Late booking for antenatal care (≥12 wk)**			0.18				
	Yes	85 (23.3)	150 (19.8)		1.23	0.91–1.66	1.08	0.77–1.50
	No	280 (76.7)	607 (80.2)		1		1	
	**Parity**			0.001				
	0	197 (54.1)	330 (43.6)		1.53	1.19–1.96	1.6	1.17–2.20
	≥1	167 (45.9)	427 (56.4)		1		1	
	**Previous cesarean delivery**			0.002				
	Yes	47 (12.9)	96 (12.7)		1.33	0.89–2.0		
	No	121 (33.2)	330 (43.7)		1			
	**Previous pregnancy problem**			0.001				
	Yes	65 (18.0)	141 (18.7)		1.31	0.90–1.90		
	No	100 (27.6)	284 (37.6)		1			
	**Multiple pregnancy**			0.036				
	Yes	10 (2.8)	8 (1.1)		2.63	1.03–6.73	2.8	0.81–9.72
	No	354 (97.3)	746 (98.9)		1		1	
	**Smoked during pregnancy**			0.103				
	Yes	99 (27.4)	173 (22.9)		1.27	0.95–1.69	1.13	0.81–1.57
	No	262 (72.6)	581 (77.1)		1		1	
	**Body mass index at booking (kg/m^2^)**			0.982				
	<18.5	15 (4.1)	29 (3.8)		1.1	0.58–2.11	0.71	0.32–1.60
	18.5<25	159 (43.6)	339 (44.8)		1		1	
	25<30	96 (26.3)	196 (25.9)		1.04	0.77–1.42	1.1	0.77–1.57
	≥30	95 (26.0)	193 (25.5)		1.05	0.77–1.43	1.2	0.83–1.74
	**Diabetes**			0.35				
	Yes	10 (2.7)	29 (3.8)		0.71	0.34–1.47	0.8	0.35–1.83
	No	355 (97.3)	728 (96.2)		1		1	
	**History of pyelonephritis/urinary tract infection**			<0.001				
	Yes	36 (9.9)	33 (4.4)		2.4	1.47–3.92	1.31	0.71–2.42
	No	329 (90.1)	724 (95.6)		1		1	
	**History of sexually transmitted infection**			0.029				
	Yes	26 (7.2)	31 (4.1)		1.8	1.05–3.09	1.63	0.91–2.90
	No	336 (92.8)	723 (95.9)		1		1	
	**Pre-existing medical problem** ****			<0.001				
	Yes	120 (32.9)	171 (22.7)		1.67	1.27–2.20	1.4	1.01–1.94
	No	245 (67.1)	583 (77.3)		1		1	
	**Invasive antenatal procedure** *****			0.57				
	Yes	5 (1.38)	11 (1.46)		0.94	0.32–2.73	0.66	0.18–2.42
	No	358 (98.6)	742 (98.5)		1		1	
	**Febrile illness or antibiotics in 2 wk before presentation**			<0.001				
	Yes	153 (41.9)	42 (5.6)		12.29	8.45–17.86	12.07	8.11–17.97
	No	212 (58.1)	715 (94.5)		1		1	

*Percentage of individuals with complete data.

**Adjusted for all factors in the table. Age treated as a continuous linear term in the analysis, but presented as a categorical term.

***As per the National Statistics Socio-Economic Classification (http://www.ons.gov.uk/ons/guide-method/classifications/current-standard-classifications/soc2010/soc2010-volume-3-ns-sec—rebased-on-soc2010—user-manual/index.html).

****Major pre-existing medical problems (percent cases versus controls) include asthma (10.0% versus 17.0%), endocrine disorders (5.8% versus 9.4%), haematological disorders (9.2% versus 7.0%), mental health/psychiatric disorders (13.3% versus 12.9%), renal disorders (7.5% versus 1.8%), and unknown medical problem (18.3% versus 15.8%).

*****Chorionic villus sampling, amniocentesis, etc.

**Table 3:** Unadjusted and adjusted odds ratios for severe sepsis associated with delivery factors in postpartum cases compared with controls.

Risk Factor	Postpartum Cases *n* (Percent)*, *n* = 231	Controls *n* (Percent)*, *n* = 757	Chi-Square *p*-Value	uOR	95% CI	aOR**	95% CI
**Premature rupture of membranes**			0.476				
Yes	21 (13.7)	74 (11.6)		1.21	0.72–2.03	0.98	0.53–1.81
No	132 (86.3)	562 (88.4)		1		1	
**>5 vaginal examinations**			0.04				
Yes	53 (23.1)	127 (17.1)		1.46	1.02–2.09	1.09	0.66–1.79
No	176 (76.9)	615 (82.9)		1		1	
**Fetal blood sampling**			0.003				
Yes	19 (8.2)	27 (3.6)		2.42	1.32–4.44	1.03	0.48–2.19
No	212 (91.8)	730 (96.4)		1		1	
**Fetal scalp electrode**			0.027				
Yes	32 (13.9)	67 (8.9)		1.66	1.06–2.60	1.14	0.64–2.06
No	199 (86.2)	690 (91.2)		1		1	
**Labour induction**			0.563				
Yes	62(26.8)	218 (28.8)		0.91	0.65–1.26	1.13	0.75–1.70
No	169 (73.2)	539 (71.2)		1		1	
**Mode of delivery**			<0.001				
Spontaneous vaginal	51 (25.5)	443 (58.8)		1		1	
Operative vaginal	29 (14.5)	100 (13.3)		2.52	1.52–4.17	2.49	1.32–4.70
Pre-labour cesarean	44 (22.0)	119 (15.8)		3.21	2.05–5.04	3.83	2.24–6.56
Cesarean after labour onset	76 (38.0)	92 (12.2)		7.18	4.72-10.92	8.06	4.65–13.97
**Complications of delivery** ***			0.46				
Yes	79 (34.2)	279 (36.9)		0.89	0.65–1.21	1.69	1.09–2.63
No	152 (65.8)	478 (63.1)		1		1	

*Percentage of individuals with complete data.

**Adjusted for all factors in the table as well as age, ethnic group, socio-economic group, parity, multiple gestation, history of urinary tract infection, pre-existing medical problems, and febrile illness or antibiotics in the 2 wk prior to presentation.

***Complications of delivery (percent cases versus controls) include episiotomy (12.3% versus 13.9%), tears (second to fourth degree) (6.5% versus 18.2%), manual removal of placenta (1.3% versus 1.5%), postpartum haemorrhage (4.3% versus 2.3%), and other complications of cesarean section (uterine angle tear, difficult delivery of infant, ureter/bladder damage, bowel perforation, multiple adhesions, other) (10.4% versus 0.7%).

### Risk Factors for Septic Shock

**Figure 3:**
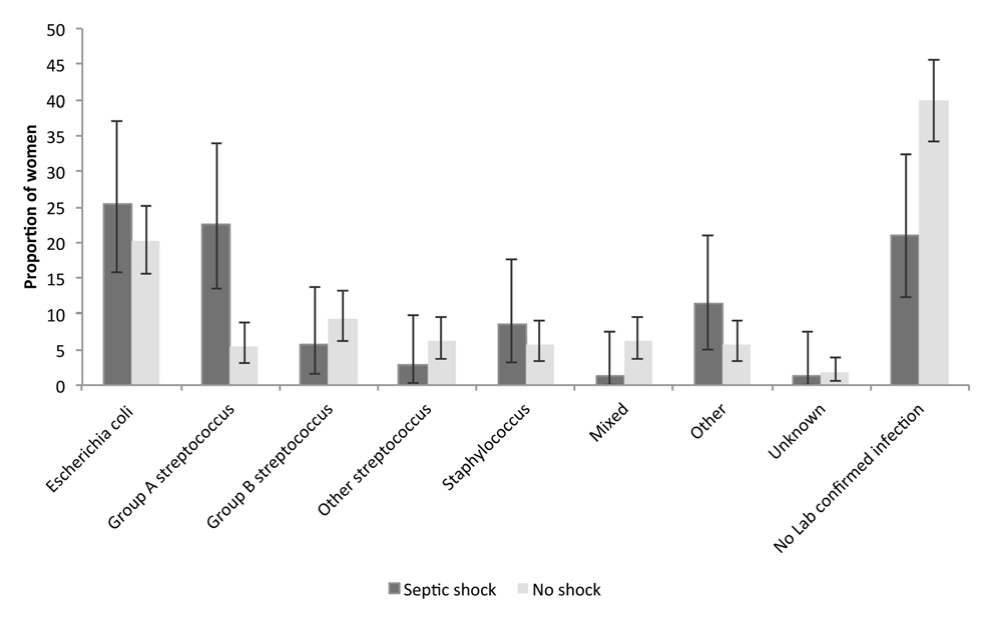
Distribution of causative organisms according to septic shock diagnosis. Bars represent the proportion, and whiskers the corresponding 95% CIs, of women with septic shock versus no shock, distributed according to causative organism.

A priori sociodemographic, infection, and delivery characteristics amongst woman who had septic shock, compared to women with severe sepsis but not septic shock, are described in Table 4 and Figure 3. After adjustment for all a priori and infection factors in the model, multiple pregnancy and group A streptococcus as the causative organism were significantly associated with an increase in the odds of progression from severe sepsis to septic shock. Before adjustment for group A streptococcal infection, spontaneous vaginal delivery (aOR = 3.85; 95% CI 1.35–10.96) and operative vaginal delivery (aOR = 3.12; 95% CI 1.03–9.57) were significantly associated with an over 3-fold increase in the odds of progression to septic shock.

**Table 4:** Unadjusted and adjusted odds for septic shock comparing cases of septic shock with cases of severe sepsis without septic shock.

Category	Risk Factor	Septic Shock *n* (Percent), *n* = 71	Severe Sepsis without Shock *n* (Percent), *n* = 294	Chi-Square *p*-Value	uOR	95% CI	aOR*	95% CI
**A priori sociodemographic and infection factors**	**Age (years)**			0.011				
	<25	16 (22.5)	101 (34.6)		1		1	
	25–34	35 (49.3)	151 (51.4)		1.46	0.77–2.78	1.25	0.60–2.58
	≥35	20 (28.2)	42 (14.3)		3.01	1.42–6.36	2.24	0.94–5.30
	**Ethnic group**			0.608				
	White	45 (63.4)	176 (60.1)		1		1	
	Black and other minority	26 (36.6)	117 (39.9)		0.87	0.51–1.48	0.77	0.42–1.38
	**Socio-economic group**			0.677				
	Unemployed	12 (16.9)	56 (19.1)		1		1	
	Employed	59 (83.1)	238 (81.0)		1.16	0.58–2.30	0.93	0.51–1.72
	**Febrile illness or antibiotics in 2 wk before presentation**			0.637				
	Yes	43 (60.6)	169 (57.5)		0.88	0.52–1.49	0.85	0.47–1.51
	No	28 (39.4)	125 (42.5)		1		1	
	**Parity**			0.002				
	0	26 (37.1)	171 (58.2)		1		1	
	≥1	44 (62.9)	123 (41.8)		2.35	1.37–4.03	1.7	0.94–3.10
	**Multiple pregnancy**			0.012				
	Yes	5 (7.1)	5 (1.7)		4.45	1.25–15.81	5.75	1.54–21.45
	No	65 (92.9)	289 (98.3)		1		1	
	**Postpartum**			0.255				
	Yes	62 (87.3)	240 (81.6)		1.55	0.73–3.31	1.02	0.45–2.33
	No	9 (12.7)	54 (18.4)		1		1	
	**Organism**			<0.001				
	*E. coli*	18 (25.4)	59 (20.1)		1			
	Group A streptococcus	16 (22.5)	16 (5.4)		3.28	1.37–7.83		
	Group B streptococcus	4 (5.6)	27 (9.2)		0.49	0.15–1.57		
	Other streptococcus	2 (2.8)	18 (6.1)		0.36	0.08–1.72		
	Staphylococcus	6 (8.5)	17 (5.8)		1.16	0.40–3.37		
	Mixed	1 (1.4)	18 (6.1)		0.18	0.02–1.46		
	Other	8 (11.3)	17 (5.8)		1.54	0.57–4.16		
	Unknown	1 (1.4)	5 (1.7)		0.66	0.07–5.98		
	No laboratory-confirmed infection	15 (21.1)	117 (39.8)		0.42	0.20–0.89		
	**Group A streptococcus organism**			<0.001				
	Yes	16 (22.5)	16 (5.4)		5.05	2.39–10.71	4.84	2.17–10.78
	No	55 (77.5)	278 (94.6)		1		1	
**Delivery factors (postpartum only)**	**Mode of delivery** **			<0.001				
	Spontaneous vaginal	21 (29.5)	36 (12.3)		5.06	2.16–11.86	2.49	0.81–7.65
	Operative vaginal	8 (11.3)	31 (10.6)		2.43	0.88–6.76	2.89	0.92–9.09
	Pre-labour cesarean	9 (12.7)	58 (19.9)		1.76	0.66–4.69	1.12	0.37–3.42
	Cesarean after labour onset	9 (12.7)	99 (33.9)		1		1	
	No delivery***	24 (33.8)	68 (23.3)					
	**Delivery complications**			0.123				
	Yes	15 (21.1)	88 (29.9)		0.71	0.36–1.37	0.67	0.33–1.39
	No	32 (45.1)	138 (46.9)		1		1	
	No delivery***	24 (33.8)	68 (23.3)					
	**Woman readmitted**			0.08				
	Yes	23 (32.4)	87 (29.6)		1.42	0.76–2.66	1	
	No	24 (33.8)	139 (47.3)		1		0.93	0.33–1.39
	No delivery***	24 (33.8)	68 (23.3)					

*Adjusted for all factors in the table.

**Before adjustment for group A streptococcus: spontaneous vaginal aOR = 3.85 (95% CI 1.35–10.96); operative vaginal aOR = 3.12 (95% CI 1.03–9.57).

***Includes antepartum cases and first/second trimester loss.

### Severe Genital Tract Sepsis

When the logistic models were re-run specifically including only cases with genital tract infection (*n* = 113) compared to controls, women who were black or from another minority ethnic group (aOR = 2.08; 95% CI 1.27–3.40), had a multiple pregnancy (aOR = 5.29; 95% CI 1.31–21.44), or had a febrile illness or were taking antibiotics in the 2 wk prior to delivery (aOR = 11.70; 95% CI 6.83–20.07) had significantly increased odds of severe sepsis. After adjusting for a priori factors, compared to women who had a spontaneous vaginal delivery, and controlling for illness prior to delivery, women who had a pre-labour cesarean section (aOR = 2.67; 95% CI 1.16–6.14), cesarean section after the onset of labour (aOR = 6.91; 95% CI 2.96–16.13), or a complication of delivery (aOR = 2.10; 95% CI 1.09–4.05) had significantly increased odds of severe sepsis. Of women with severe genital tract sepsis, 27 (23.9%) developed septic shock. Infection with group A streptococcus (aOR = 3.30; 95% CI 1.03–10.53) was the single factor associated with an increased odds of septic shock.

## Discussion

We found that for each maternal sepsis death in the UK, approximately 50 women have life-threatening morbidity from sepsis, and the onset of severe sepsis from SIRS occurs very rapidly. Genital tract and urinary tract infections are the predominant sources of infection; all modes of operative delivery carry significant risks for severe sepsis; and whilst the largest proportion of cases of severe sepsis is caused by *E. coli*, outcomes are significantly worse for women with group A streptococcal infection. Importantly, women who are treated with antibiotics in the perinatal period are at significant risk of severe sepsis, suggesting that a significant proportion of infections progress even following antibiotic treatment. These findings highlight a number of key messages for clinical practice in both primary and secondary care, with the high levels of life-threatening morbidity identified indicating that pregnant or recently pregnant women with suspected infection need closer attention than women who are not pregnant.

Strengths of this study include the robust design and participation of 100% of the maternity units in the UK, thus many limitations concerning regional differences, population size, and selection bias were minimised. It is possible that some women with severe sepsis in pregnancy were not admitted to maternity units, and thus not included in the study population. However, in the majority of cases, an obstetrician would be consulted about the care of such women, and these patients are thus likely to be brought to the attention of maternity services. In addition, while the distribution of antepartum sepsis cases is in keeping with the UK Confidential Enquiry into Maternal Deaths, with the majority of sepsis deaths occurring later in pregnancy [2], since UKOSS data is collected from maternity units it may be that first trimester cases were under-captured; however, it was not possible to audit this. Lastly, results of the distribution of causative organisms were limited by the proportion of women with a clinical diagnosis of sepsis, but no identified organism. Failure to identify a causative organism in a proportion of cases is, however, to be expected [15] and therefore may not be regarded as a limitation, given that there is currently no other UK study that has elucidated the distribution of causative organisms for severe maternal sepsis.

The incidence rate and risk factors identified concur with previous studies of severe maternal sepsis [1],[2],[4],[19],[20], and the results are likely to be generalisable to other high-resource settings such as the US and the Netherlands, which have experienced similar increases in severe maternal morbidity and mortality from sepsis [3],[4],[21]. A recent national study in the US found that maternal mortality from sepsis increased by 10% per year from 1998 to 2008 [21], and another large population-based cohort study in the US found that the incidence of severe maternal sepsis in 2005–2007 was nearly double the 2003 estimate [4]. In addition, risk factors identified in our study, such as black or other minority ethnic group, primiparity, and multiple pregnancy, were also identified in the two US-based studies. These similarities suggest that our findings have generalisable implications for clinical practice, guideline development, and further study of causative organisms. Many clinical messages relate to basic care and can also be generalised to obstetric services in lower-resource countries. The limitations that apply to all case-control studies using multivariable analysis also apply to this study, and the level of evidence should be considered on this basis.

With further regards to incidence rates, Waterstone and colleagues, in the only other large population-based study of severe maternal sepsis in the UK, reported an incidence of 4.0 (95% CI 2.0–6.0) per 10,000 maternities in southwest England during the period from 1997 to 1998 [1]. The incidence of 4.7 per 10,000 maternities identified in the current study represents a 15% increase, which corresponds to the increase in maternal deaths from sepsis in the UK since this period (0.85 to 1.13 per 100,000 maternities [2]). An incidence of 4.7 is also within the range of other population-based studies of severe maternal sepsis, most recently 2.1 per 10,000 in the Netherlands [20], 2.1 per 10,000 in Scotland [19], and 4.9 per 10,000 in the US [4]. It is interesting to note that incomplete information (and thus underreporting) was discussed as a limitation of the Dutch study [20]; it is possible, therefore, that the rate in the Netherlands might be closer to that found in this study.

Severe sepsis in pregnancy presents in primary care, and the previously undescribed association between antibiotic prescription in the perinatal period and risk of severe sepsis suggests that primary care practitioners should have a low threshold for referral of women in pregnancy with signs of infection. Over 40% of women with severe sepsis had a febrile illness or were taking antibiotics prior to presentation, which suggests that at least a proportion were not adequately diagnosed, treated, or followed up. It cannot be assumed that antibiotics will prevent progression to severe sepsis, and safety net checks—for example, follow-up appointments or instructions to return if symptoms do not resolve—should therefore be in place to make sure a pregnant woman treated for infection has recovered. Simply prescribing antibiotics alone may not be appropriate. This message applies equally to secondary care; there is a need to ensure that follow-up happens to ensure that treatment is effective.

As sepsis progresses along a spectrum of severity, the occurrence of life-threatening sepsis represents the severest end short of a maternal death, and therefore only the “tip of the iceberg” of serious maternal morbidity. Failure to recognise the severity of an infection is a ubiquitous factor in the progression to severe sepsis [2],[22],[23]. Intensivists have the most training in sepsis management; however, initial presentation is often to general practitioners or to accident and emergency medical staff with less awareness of the signs and symptoms of sepsis, or of the rapidity with which it may progress to severe sepsis in the obstetric population [24]. In our study population, for most women with severe sepsis there was less than 24 h between the first sign of SIRS and the diagnosis of severe sepsis, and for most women with a group A streptococcal infection there was less than 9 h between the first sign of SIRS and severe sepsis, with half having less than 2 h between the first signs and diagnosis.

The rapid progression to severe sepsis highlights the importance of following the international Surviving Sepsis Campaign's guidelines in pregnancy, and the recommendation for administration of high-dose intravenous antibiotics within 1 h of admission for anyone with suspected sepsis [25].

A challenge in all previous studies of maternal sepsis has been to assess the temporality of mode of delivery in relation to infection and sepsis. Our study shows that after controlling for illness before delivery, as well as clinical risk factors such as premature rupture of membranes, all modes of operative delivery (operative vaginal, pre-labour cesarean, and cesarean after the onset of labour) were independent risk factors for severe sepsis. Even though antibiotic prophylaxis at cesarean section is routine practice in the UK, these results suggest that women are still at heightened risk of severe sepsis, despite the administration of antibiotics, and emphasise the importance of attention to prophylaxis particularly in emergency deliveries. The risk associated with operative vaginal delivery confirms findings from a previous study [19], and suggests there is a need for further investigation of the role of prophylactic antibiotics as well as stringent attention to infection control measures for these deliveries.

The different patterns of infection we observed in antenatal and postnatal women suggest that overall greater consideration needs to be given to the source of infection, and therefore the most appropriate antibiotic to prescribe. This study highlights that urinary tract infection remains an important cause of severe sepsis, particularly antenatally, so prompt treatment and follow-up in primary care to ensure that the infection is eradicated is important. This finding was not identified in the most recent UK Confidential Enquiry into Maternal Deaths [2], and provides further evidence of the importance of investigation of severe morbidity as well as mortality in high-resource settings to generate actions to prevent severe disease.

Our results indicate that although severe sepsis is more common following cesarean delivery, women delivering vaginally are at heightened risk of group A streptococcal infection, and those that are infected with group A streptococcus are at significantly increased risk of progression to septic shock compared with women infected with another organism. These results are consistent with the recent trend in maternal sepsis deaths in the UK; 50% of direct genital tract sepsis deaths in the most recent Confidential Enquiry into Maternal Deaths were caused by group A streptococcus [2]. Correspondingly, 50% of proven group A streptococcal infections in our study population led to septic shock, with very rapid progression from the first sign of SIRS. This has a direct implication for decisions about the availability of rapid antigen diagnostic tests for group A streptococcus in obstetrics. While culture remains the gold standard for confirmation of group A streptococcus, it takes 1–2 d to obtain results, which is significantly longer than the time course from the first signs of SIRS to septic shock for most women. In the absence of rapid diagnostics, a positive culture for group A streptococcus should be reported urgently by telephone as soon as it is discovered in the laboratory, and prior to this, a clinical suspicion of group A streptococcus should be regarded as a red flag for urgent action and very close monitoring. In addition, training about group A streptococcal infection should be routinely included in all obstetric emergency training courses.

In conclusion, this study emphasises that both primary and secondary care practitioners should remain aware that pregnant or recently pregnant women with suspected infection need closer attention than women who are not pregnant. Antibiotic prescription does not necessarily prevent progression to severe sepsis, and women should be followed up to ensure recovery. The rapid progression to severe sepsis highlights the importance of following the international Surviving Sepsis Campaign guideline of administration of high-dose intravenous antibiotics within 1 h of admission to hospital for anyone with suspected sepsis. Signs of severe sepsis, particularly with confirmed or suspected group A streptococcal infection, should be regarded as an obstetric emergency and should be routinely included in obstetric emergency training courses. Consideration could be given to a change of timing of prophylactic antibiotics to administration at time of decision for emergency cesarean section, and vigilant infection control at vaginal delivery should be maintained, with a potential role for prophylactic antibiotics at operative vaginal delivery. Future research should assess the efficacy of rapid antigen diagnostic tests for group A streptococcus in obstetrics.

## Supporting Information

Checklist S1 **Strobe checklist.**
(DOC)

## Abbreviations

aOR: adjusted odds ratio
IQR: interquartile range
OR: odds ratio
SIRS: systemic inflammatory response syndrome
UKOSS: United Kingdom Obstetric Surveillance System
uOR: unadjusted odds ratio
